# Efficacy of tranexamic acid in patients with traumatic brain injury

**DOI:** 10.17179/excli2020-3161

**Published:** 2020-12-08

**Authors:** Tomoyuki Kawada

**Affiliations:** 1 Department of Hygiene and Public Health, Nippon Medical School

## ⁯⁯

***Dear Editor,***

Several results have been reported on the efficacy of tranexamic acid (TXA) in patients with acute traumatic brain injury (TBI). As such, Lawati et al. (2020[4]) conducted a meta-analysis to determine the safety and efficacy of TXA in these patients. The authors summarized nine randomized controlled trials (RCT), which resulted in a pooled relative risk (RR) (95% confidence interval [CI]) of TXA of 0.95 (0.88-1.02) for mortality. There was also no significant difference in disability assessed using the Disability Rating Scale. Furthermore, the pooled RR (95% CI) of TXA for hematoma expansion was 0.77 (0.58-1.03). Although the use of TXA did not increase the risk of adverse events, no advantages were observed for using TXA in TBI patients.

Rowell et al. (2020[5]) conducted a double-blinded RCT to determine whether TXA treatment initiated in the out-of-hospital setting within 2 hours of injury improved neurologic outcome in patients with moderate or severe TBI. Similarly, TXA administration did not improve the 6-month neurologic outcome as measured using the Glasgow Outcome Scale-Extended. Cone et al. (2020[1]) also commented that a large trial assessing efficacy with optimized dosing protocols, a mortality end point, and specific focus on the TBI severity cohorts should be conducted to verify the existence of benefits.

In contrast, an RCT report presented that the adjusted RRs (95% CIs) of TXA for mild-to-moderate and severe TBI death were 0.78 (0.64-0.95) and 0.99 (0.91-1.07), respectively (CRASH-3 trial collaborators, 2019[2]). In addition, early treatment was more effective than later treatment in patients with mild-to-moderate head injury that could result in death. This means that the level of severity in patients with TBI is important for subsequent clinical interventions.

There is a comment regarding the inconsistent results (Kawada, 2020[3]), and a meta-analysis of RCTs ensuring high-quality of each trial should be conducted with special reference to the level of severity.

## Conflict of interest

The author declares no conflict of interest.

## References

[1] Cone DC, Spaite DW, Coats TJ (2020). Out-of-hospital tranexamic acid for traumatic brain injury. JAMA.

[2] CRASH-3 trial collaborators (2019). Effects of tranexamic acid on death, disability, vascular occlusive events and other morbidities in patients with acute traumatic brain injury (CRASH-3): a randomised, placebo-controlled trial. Lancet.

[3] Kawada T (2020). The efficacy of tranexamic acid for brain injury. Am J Emerg Med.

[4] Lawati KA, Sharif S, Maqbali SA, Rimawi HA, Petrosoniak A, Belley-Cote EP (2020). Efficacy and safety of tranexamic acid in acute traumatic brain injury: A systematic review and meta-analysis of randomized-controlled trials. Intensive Care Med.

[5] Rowell SE, Meier EN, McKnight B, Kannas D, May S, Sheehan K (2020). Effect of out-of-hospital tranexamic acid vs placebo on 6-month functional neurologic outcomes in patients with moderate or severe traumatic brain injury. JAMA.

